# Why can Mozambique Tilapia Acclimate to Both Freshwater and Seawater? Insights From the Plasticity of Ionocyte Functions in the Euryhaline Teleost

**DOI:** 10.3389/fphys.2022.914277

**Published:** 2022-05-31

**Authors:** Mayu Inokuchi, Junya Hiroi, Toyoji Kaneko

**Affiliations:** ^1^ Department of Aquatic Bioscience, Graduate School of Agricultural and Life Sciences, the University of Tokyo, Bunkyo, Japan; ^2^ Department of Anatomy, St. Marianna University School of Medicine, Kawasaki, Japan

**Keywords:** ionocyte, tilapia, osmoregulation, classification, plasticity

## Abstract

In teleost fishes, ionocytes in the gills are important osmoregulatory sites in maintaining ionic balance. During the embryonic stages before the formation of the gills, ionocytes are located in the yolk-sac membrane and body skin. In Mozambique tilapia embryos, quintuple-color immunofluorescence staining allowed us to classify ionocytes into four types: type I, showing only basolateral Na^+^/K^+^-ATPase (NKA) staining; type II, basolateral NKA and apical Na^+^, Cl^−^ cotransporter 2; type III, basolateral NKA, basolateral Na^+^, K^+^, 2Cl^−^ cotransporter 1a (NKCC1a) and apical Na^+^/H^+^ exchanger 3; and type IV, basolateral NKA, basolateral NKCC1a and apical cystic fibrosis transmembrane conductance regulator Cl^−^ channel. The ionocyte population consisted mostly of type I, type II and type III in freshwater, while type I and IV dominated in seawater. In adult tilapia, dual observations of whole-mount immunocytochemistry and scanning electron microscopy showed morphofunctional alterations in ionocytes. After transfer from freshwater to seawater, while type-II ionocytes closed their apical openings to suspend ion absorption, type-III ionocytes with a concave surface were transformed into type IV with a pit *via* a transitory surface. The proposed model of functional classification of ionocytes can account not only for ion uptake in freshwater and ion secretion in seawater, but also for plasticity in ion-transporting functions of ionocytes in tilapia.

## Introduction

Osmoregulation is a physiological mechanism to maintain a stable internal environment which is required for optimal cell function. In teleost fishes, although they inhabit various osmotic environments, their plasma osmolality is maintained within narrow physiological ranges, equivalent to about one-third seawater (SW) osmolality. Osmoregulation in adult teleosts largely consists of integrated ion and water transport activities of the gills, kidney and intestine. In particular, ionocytes, previously referred to as chloride cells or mitochondrion-rich cells, in the gills are important osmoregulatory sites in maintaining ionic balance (Hwang and Lin, 2013; Kültz and Gilmour, 2020). Ionocytes are responsible for ion uptake in freshwater (FW) and ion secretion in SW. Ionocytes in the gills are also known as a multifunctional cell, which is the dominant site for acid-base regulation and nitrogenous waste excretion in addition to osmoregulation. As is the case in adult fish, teleost embryos and larvae are also able to maintain the osmotic balance of their body fluid (Guggino 1980; Kaneko et al., 1995; Varsamos et al., 2005; Brauner and Rombough 2012). During the early life stages of fishes, a rich population of ionocytes are present in the yolk sac membrane and body skin as a main osmoregulatory site (Lasker and Threadgold 1968; Guggino 1980; Hwang and Hirano 1985; Hwang et al., 1999). In early studies on ionocytes, electron microscopic observations and molecular identification of ion transporters have demonstrated the difference in morphology and function between FW and SW. However, it had not been elucidated how multiple ion transporters cooperate in ionocytes and how the cells switch their function following environmental salinity change. In this review, we describe the ionocyte classification and their functional plasticity between FW and SW during early life and adult stages of Mozambique tilapia (*Oreochromis mossambicus*).

Tilapia, the genus *Oreochromis*, are widely distributed in tropical areas in the wild and cultivated in fish farms owing to their hardy nature, rapid growth rates, and tolerance to varied environmental salinity (Pullin 1991). Among those tilapia species, Mozambique tilapia *O. mossambicus* is one of the most suitable fish species for studies on osmoregulation, because this euryhaline tilapia is tolerant not only to a wide range of salinity from FW to SW but also to extremely low-ion water and even to double-strength SW (Stickney, 1986; Uchida et al., 2000; Fiess et al., 2007; Inokuchi et al., 2008). Mozambique tilapia is also known as a maternal mouth brooder, in which the female incubates the fertilized eggs in her mouth, and its embryos are available all year around. Furthermore, they can breed either in FW or in SW, and embryos develop normally in respective media. The tilapia embryos are also able to survive direct transfer from FW to SW and *vice versa*. Their strong euryhalinity during both early life and adult stages intrigued us to explore the mechanisms of osmoregulation and salinity tolerance.

## Ionocyte Classification of Yolk-Sac Membrane and Body Skin in Tilapia Embryos

During the embryonic stages before the formation of the functional gills and kidney, ionocytes have been detected in the epithelia covering the yolk and body in several teleost species (Kaneko et al., 2008). The yolk-sac membrane and body skin of embryos and larvae are structurally simple, and those ionocytes would be observable more easily than branchial ionocytes. Thus, those cutaneous ionocytes could serve as an excellent model to investigate the functions and morphology of ionocytes. Hwang and Hirano (1985) found notable differences in the intercellular organization and tight-junction structure of ionocytes in the yolk-sac membrane between FW- and SW-acclimated teleost embryos. It was also reported that, after direct transfer of tilapia larvae from FW to SW, the ionocytes markedly increased their cell size, being accompanied by accessory cells (Ayson et al., 1994; Shiraishi et al., 1997). On the other hand, ionocyte density in the yolk-sac membrane did not vary appreciably between FW and SW larvae (Ayson et al., 1994).

These morphological observations suggest the occurrence of distinct FW- and SW-type ionocytes in the yolk-sac membrane of tilapia embryos and larvae, as is the case with gill ionocytes in adult fish. However, it was not elucidated whether FW-type ionocytes are replaced by newly-differentiated SW-type ionocytes after transfer from FW to SW, or whether preexisting FW ionocytes are transformed into SW ionocytes.

By observing *in vivo* sequential changes in individual ionocytes in the yolk-sac membrane of tilapia embryos and larvae, Hiroi et al. (1999) succeeded in following morphological changes in ionocytes during acclimation to different salinities. In this study, ionocytes were vitally stained with DASPEI, a fluorescent dye specific for mitochondria, and each individual ionocyte was sequentially observed for 4 days under a confocal laser scanning microscope. The sequential observation revealed that 75% of skin ionocytes survived for 96 h after transfer from FW to SW. Moreover, the ionocytes showed a remarkable increase in size after transfer, while the size did not change in embryos and larvae kept in FW. These findings suggest that FW-type small ionocytes possess the ability to survive after direct transfer to SW and to be transformed into SW-type large ionocytes (Hiroi et al., 1999).

In addition to the crude classification of ionocytes into FW and SW types, Hiroi et al. (2008) have proposed a more detailed functional classification of ionocytes. Ion-transporting functions of ionocytes are defined by various ion transporters located at either basolateral or apical membranes. Since Na^+^ and Cl^−^ are the major electrolytes in plasma, occupying more than 90% of inorganic electrolytes, they focused on transporters in charge of Na^+^ and Cl^−^ transport among various transporters expressed in ionocytes. Among those ion transporters, Na^+^/K^+^-ATPase (NKA) located at the basolateral membrane is universally present in ionocytes, providing the driving force for ion transport (Hwang and Lin, 2013; Kültz and Gilmour, 2020). The well accepted model of ion secretion in SW-type ionocytes is mediated by NKA and Na^+^, K^+^, 2Cl^−^ cotransporter 1a (NKCC1a) in the basolateral membrane, and by the cystic fibrosis transmembrane conductance regulator (CFTR) Cl^−^ channel in the apical membrane (Marshall, 2011; Kültz and Gilmour, 2020). By contrast, the mechanisms for ion uptake in ionocytes of FW-acclimated teleosts vary according to species (Dymowska et al., 2012). Therefore, the research on detailed molecular mechanisms of FW ionocytes was required across a wide range of teleost species.

To detect NKCC in ionocytes, mouse monoclonal antibody against human colonic NKCC1 (named T4; available from the Developmental Studies Hybridoma Bank; Lytle et al., 1995) has been widely and repeatedly used in various teleost species. However, the immunoreaction with T4 antibody was unexpectedly detected in the apical and basolateral membranes of FW- and SW-type ionocytes, respectively, in Mozambique tilapia (Wu et al., 2003; Hiroi et al., 2005), mummichog (Katoh et al., 2008), Japanese medaka (Hsu et al., 2014), European sea bass (Lorin-Nebel et al., 2006) and Japanese sea bass (Inokuchi et al., 2017). This phenomenon implied that two different cation–chloride cotransporters existed in ionocytes: apically-located, ion-absorptive “FW-type” and basolaterally-located, ion-secretory “SW-type”. By immunoscreening of a cDNA expression library with T4 antibody, Hiroi et al. (2008) obtained cDNA of tilapia Na^+^, Cl^−^ cotransporter 2 (NCC2) in addition to NKCC1a, NKCC1b and NKCC2. Among the four cation-chloride cotransporter candidates, the mRNA of NKCC1a was highly expressed in the yolk-sac membrane and the gills of SW-acclimated fish, whereas the NCC2 expression was restricted to those of FW fish.

**FIGURE 1:**
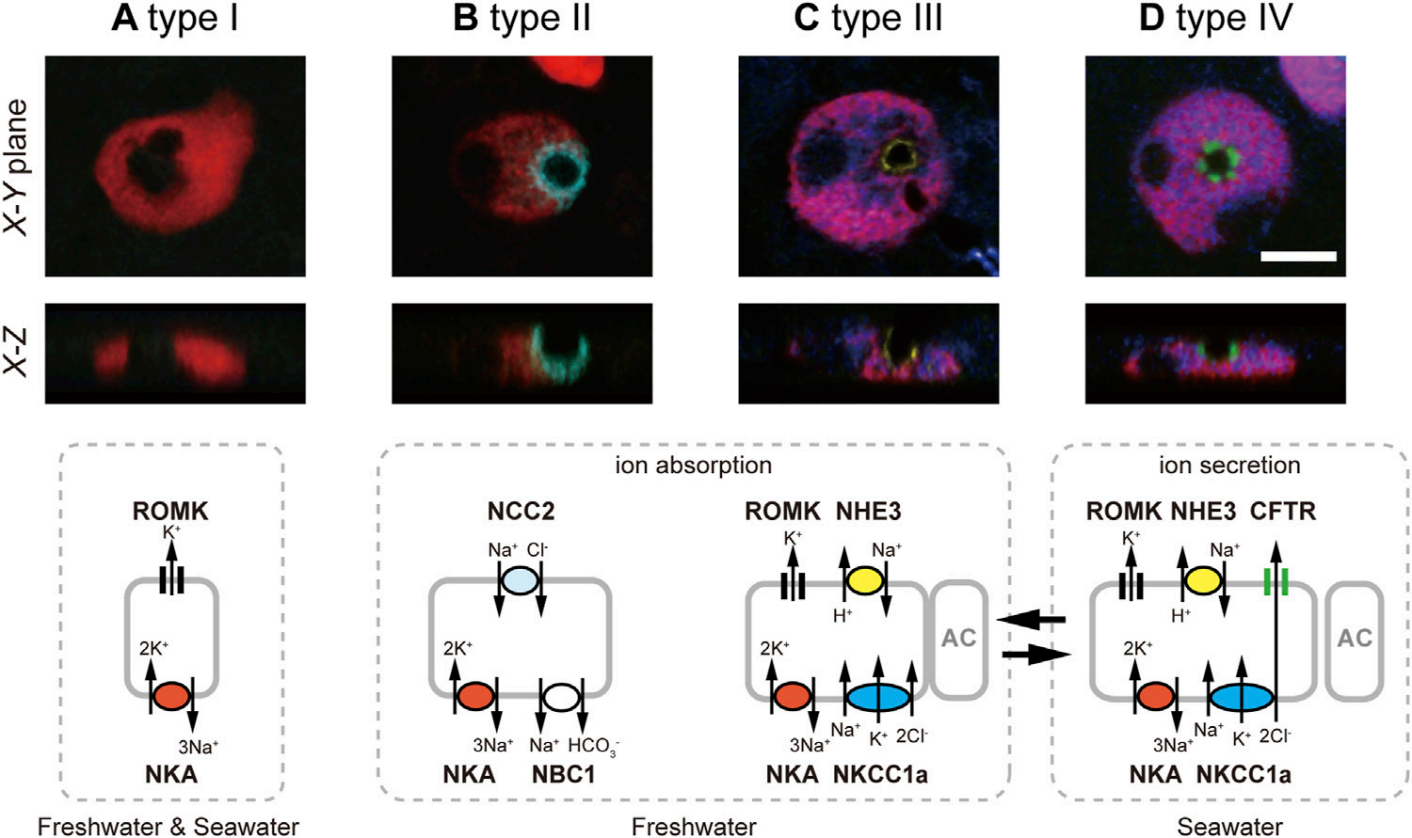
Four types of ionocytes in the yolk-sac membrane of Mozambique tilapia embryos, by means of quintuple-color immunofluorescence staining: type-I **(A)**, type-II **(B)**, type-III **(C)** and type-IV **(D)**. Five images for NKA (red), NKCC1a (blue), NCC2 (cyan), NHE3 (yellow) and CFTR (green) are merged and shown in X–Y and X–Z planes. Type-I ionocytes show only basolateral NKA. Type-II ionocytes possess basolateral NKA and distinct apical NCC2. Type-III ionocytes are defined by basolateral NKA, basolateral NKCC1a (red for NKA and blue for NKCC1a are merged into magenta), and apical NHE3. Type-IV ionocytes are provided with the three major ion-transporting proteins for salt secretion, basolateral NKA, basolateral NKCC1a and apical CFTR. Scale bar, 10 μm. Schematic diagrams of the 4 cell types are presented in the bottom row, showing the apical or basolateral localization patterns of NKA (red), NKCC1a (blue), NCC2 (cyan), NHE3 (yellow) CFTR (green), NBC1 and ROMK. Modified from Hiroi et al. (2008).

Generating antibodies specific for tilapia NKCC1a and NCC2, Hiroi et al. (2008) conducted whole-mount immunofluorescence staining for NKCC1a and NCC2, together with NKA, CFTR and Na^+^/H^+^ exchanger 3 (NHE3), on the yolk-sac membrane of tilapia embryos acclimated to FW or SW. The powerful quintuple-color immunofluorescence staining allowed them to successfully classify ionocytes into four distinct types: type I, showing only basolateral NKA staining; type II, basolateral NKA and apical NCC2; type III, basolateral NKA, basolateral NKCC1a and apical NHE3; and type IV, basolateral NKA, basolateral NKCC1a and apical CFTR (Figure 1). The type-I ionocyte is relatively small in size, showing only basolateral NKA staining. Therefore, it was first suggested as an immature ionocyte (Hiroi et al., 2005). However, this type was later considered to be an independent functional cell type (Hiroi et al., 2008), and one possible function is K^+^ secretion through renal outer medullary K^+^ channel (ROMK) found at the apical membrane (Furukawa et al., 2014). The type-II ionocyte possessing basolateral NKA and apical NCC2 is specific to FW, and the apical NCC2 is considered as a pathway to absorb Na^+^ and Cl^−^ (Hiroi et al., 2008). Type-III ionocyte defined by basolateral NKA and basolateral NKCC1a and apical NHE3 rarely appeared in SW, rapidly increased in number following transfer from SW to FW and disappeared following transfer from FW to SW. Type IV represents a typical distributional pattern of ion transporters for NaCl-secretory ionocytes in SW teleost fish. This type possesses basolateral NKA and basolateral NKCC1a similar to type-III ionocytes, but CFTR is located in the apical membrane instead of NHE3. Furthermore, the type-IV ionocyte was not observed in FW fish, but rapidly increased in number after SW transfer and disappeared after transfer back into FW (Hiroi et al., 2008). This inverse relationship between type III and IV suggests that those ionocytes have the same cell linage but transform reversibly into each other during environmental salinity changes. The proposed model of functional classification of ionocytes can account not only for ion uptake in FW and ion secretion in SW, but also for plasticity in ion-transporting functions of ionocytes in the euryhaline tilapia.

## Branchial Ionocytes in Adult Tilapia

The classification of ionocytes proposed in embryos turned out to be applicable to that in adult fish. Subsequent studies in adult gills showed that Na^+^, HCO_3_^-^ cotransporter 1 (NBC1) and ROMK were also localized in branchial ionocytes of Mozambique tilapia (Furukawa et al., 2011; Furukawa et al., 2014, Figure 1). The basolateral NBC1 of type-II ionocytes is likely to function as exit of Na^+^ absorbed by apical NCC2 (Furukawa et al., 2011). On the other hand, ROMK was detected at the apical membrane of types I, III and IV (Furukawa et al., 2014). As ROMK was upregulated after transfer to high-K^+^ FW or SW, ROMK seems to play an important role in K^+^ excretion in those ionocytes (Furukawa et al., 2014).

In earlier studies, scanning (SEM) and transmission (TEM) electron microscopy has been used to identify and quantify functional ionocytes including FW and SW subtypes in tilapia as in other teleost species (Pisam et al., 1995; Lee et al., 1996; van der Heijden et al., 1999). While the apical membrane of SW ionocytes typically forms a pit structure, the apical surface of FW ionocytes often appears as a flat or slightly projecting disk (Lee et al., 1996). In SEM observations, three subtypes of ionocytes were exhibited in FW tilapia: wavy convex, shallow-basin, and deep-hole (Lee et al., 1996). The apical features of deep-hole ionocytes were narrow, deep, round-to-oval pores in which little or no internal structure was visible by SEM. The wavy-convex ionocytes are characterized by a wide apical opening and a rough surface appearance. The ovoid apertures of shallow-basin ionocytes were occasionally ornamented with short microvilli. Following transfer from artificial high-Cl^−^ to low-Cl^−^ media, the wavy-convex cells were rapidly increased, while in turn the deep-hole ionocytes disappeared (Chang et al., 2003). In contrast, low-Ca^2+^ fish developed more shallow-basin ionocytes in the gills and a higher Ca^2+^ influx than those acclimated to other media (Chang et al., 2001). These findings indicated that the shallow-basin and wavy-convex ionocytes are mainly responsible for the uptake of Ca^2+^ and Cl^−^, respectively. Following transfer of tilapia from FW to brackish water (20 ppt), the wavy-convex ionocytes disappeared within 3 h, but deep-hole ones increased from 48 h (Wang et al., 2009). The rapid disappearance of wavy convex ionocytes might be due to the internalization of the apical membrane (Lin and Hwang, 2004). The morphological changes in apical surface post transfer indicates that different phenotypes play different roles in ion regulation. However, the evident relationship between the variable morphology of apical surfaces and the localization pattern of ion-transporting proteins was not clarified.

In tilapia acclimated to hypoosmotic environments, a direct comparison between SEM image and the distribution of ion transporters demonstrated the relationship between the apical membrane structures and cellular function of ionocytes (Inokuchi et al., 2009). While both NHE3 (type-III) and NCC2 (type-II) ionocytes possess small apical openings in normalized artificial FW, the NHE3 and NCC2 were confined to concave and convex apical surfaces, respectively, in lower ion conditions. The ionocytes with apical NHE3 enlarged their concave apical surface in low Na^+^ media to facilitate Na^+^ absorption. In contrast, under low Cl^−^ condition, apical NCC2 cells developed a large apical convex surface to activate Na^+^ and Cl^−^ uptake (Inokuchi et al., 2009; Choi et al., 2010).

**FIGURE 2:**
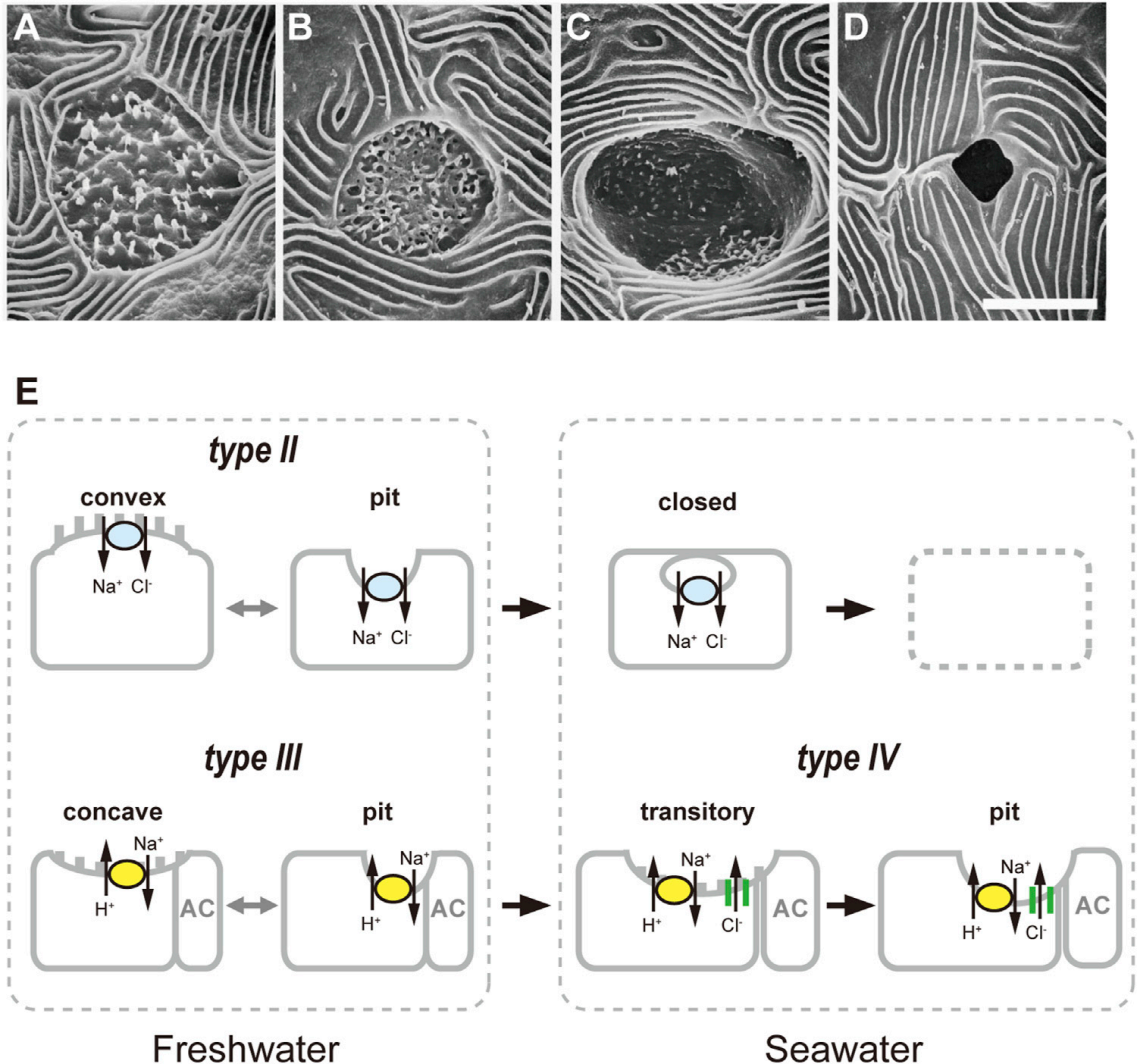
Classification of apical openings of ionocytes in the gills of Mozambique tilapia. Four types of apical openings, identified by scanning electron microscopy: a convex apical surface **(A)**, a concave apical surface **(B)**, a transitory apical surface **(C)** and an apical pit **(D)**. Scale bar, 5 μm. **(E)**, Schematic diagrams of relationship between apical opening structure and ionocyte types. The type-II ionocytes with convex apical surfaces or apical pits suspend ion-absorptive function by closing the apical openings as a quick response after transfer from freshwater to 70% seawater, followed by cell disappearance. After transfer, concave apical surfaces or small apical pits typically seen in type-III ionocytes are transformed into enlarged apical pits in type-IV ionocytes *via* the transitory apical surfaces. Modified from Choi et al. (2011).

In order to further clarify the functional and morphological response of ionocytes to acute salinity change, Choi et al. (2011) developed a new technique, dual observations of whole-mount immunocytochemistry and SEM. In this method, the gill filaments were first subjected to triple immunofluorescent staining for NKA, NHE3 and NCC2/NKCC1 (T4 antibody) to identify the types of ionocytes, followed by SEM observation on the apical structures of ionocytes in the same specimens. The comparison of the fluorescent and SEM images enables to link the apical structure to a certain ionocyte type. To examine the short-term responses of ionocytes during acclimation to different salinity environments, tilapia were directly transferred from a hypoosmotic environment (FW) to a hyperosmotic environment (70% SW). In addition to a pit surface (deep-hole type), a convex apical surface (wavy-convex type) and a concave apical surface (shallow-basin type), the SEM observation identified a transitory apical surface between the pit and concave structures (Figures 2A–D). In FW-acclimated tilapia gills, as previously reported, three types of apical openings were observed; that is, pit, convex and concave surfaces. At 6 h after transfer to 70% SW, there appeared the transitory type that was not observed in FW fish. The occurrence of the transitory apical surface that shared morphological characteristics of both a concave apical surface and an apical pit suggests a possible transformation from a concave apical surface to an apical pit. The dual observations of whole-mount immunocytochemistry and SEM then revealed that concave apical surfaces typically seen in type-III ionocytes were transformed into enlarged apical pits in type-IV (SW-type) ionocytes *via* the transitory apical surfaces. It should be noted that this dual-observation technique also revealed the occurrence of ionocytes with a closed apical surface, which was completely covered with adjacent pavement cells. The type-II ionocytes, the other FW-type cells with apical NCC2, possess a small apical pit or a convex apical opening in FW. After transfer of tilapia to 70% SW, type-II ionocytes suspended ion-absorptive function by closing the apical surfaces as a quick response to the salinity change. Interestingly, whereas type-III and type-IV ionocytes show functional plasticity to switch ion-transporting functions between FW and SW, type-II ionocytes are specific for FW acclimation (Figure 2E).

The functional plasticity of ionocytes has also been reported in other teleost species. The scanning ion-selective electrode technique (SIET) demonstrated the functional change from ion uptake to ion secretion in individual ionocytes in the skin of medaka larvae during acute salinity change (Shen et al., 2011). In Japanese sea bass, the immunocytochemical observation revealed the occurrence of intermediate-type between type-III and type-IV ionocytes at 1 day after transfer from SW to hypoosmotic environment (10% SW) (Inokuchi et al., 2017). The intermediate-type ionocytes showed apical CFTR and NHE3 immunoreactions, which are characteristic of type-IV cells; however, their apical region showing a convex appearance is more like type-III ionocytes.

## Conclusion

In this review, in order to address the mechanism of functional switching between hyper- and hypo-osmoregulation in euryhaline teleosts, we describe the ionocyte classification of Mozambique tilapia and their functional plasticity between FW and SW. In tilapia embryos, quintuple-color immunofluorescence staining allowed us to classify ionocytes into four types (Hiroi et al., 2008). In adult tilapia, dual observations of whole-mount immunocytochemistry and SEM showed morphofunctional alterations in ionocytes (Choi et al., 2011). Our findings indicate functional plasticity of type-III/IV ionocytes to switch their ion-transporting functions, whereas type-II ionocytes are considered to be specific for FW acclimation. Our morphological and functional analyses revealed how ionocytes switch their function between FW and SW, but the biological pathway to regulate functions of ionocytes is yet to be elucidated. Further studies are required, focusing not only on the ion-transporting function of ionocytes, but also on the molecular pathway regulating the functional plasticity of ionocytes. For example, the utilization of the state-of-the-art molecular approaches, such as the single-cell RNA sequencing, may provide a better understanding of this issue.

Salinity is regarded as one of the most important physical characteristics of the aquatic environment that govern the distribution of species in nature. Mozambique tilapia is a euryhaline teleost with a worldwide tropical distribution, originating from estuaries and near-shore rivers from the lower Zambezi River to the southeast coast of South Africa (Trewevas, 1983). The capacity of ionocytes to alter their morphology and ion-transporting function, or the morphofunctional plasticity of ionocytes, account at least in part for their strong salinity tolerance.
